# Association of patient photographs and reduced retract-and-reorder events

**DOI:** 10.1093/jamiaopen/ooae042

**Published:** 2024-07-02

**Authors:** Daniel Rzewnicki, Atul Kanvinde, Scott Gillespie, Evan Orenstein

**Affiliations:** Department of Medicine, Emory University School of Medicine, Atlanta, GA 30322, United States; Information Technology, Shepherd Center, Atlanta, GA 30305, United States; Department of Pediatrics, Emory University School of Medicine, Atlanta, GA 30322, United States; Department of Pediatrics, Emory University School of Medicine, Atlanta, GA 30322, United States; Information Services and Technology, Children’s Healthcare of Atlanta, Atlanta, GA 30329, United States

**Keywords:** electronic health records, wrong-patient order entry, retract-and-reorder, computerized provider order entry

## Abstract

**Background:**

Wrong-patient order entry (WPOE) is a potentially dangerous medical error. It remains unknown if patient photographs reduce WPOE in the pediatric inpatient population.

**Materials and Methods:**

Order sessions from a single pediatric hospital system were examined for retract-and-reorder (RAR) events, a surrogate WPOE measure. We determined the association of patient photographs with the proportion of order sessions resulting in a RAR event, adjusted for patient, provider, and ordering context.

**Results:**

In multivariable analysis, the presence of a patient photo in the electronic health record was associated with 40% lower odds of a RAR event (aOR: 0.60, 95% CI: 0.48-0.75), while cardiac and ICU contexts had higher RAR frequency (aOR: 2.12, 95% CI: 1.69-2.67 and 2.05, 95% CI: 1.71-2.45, respectively).

**Discussion and Conclusion:**

Patient photos were associated with lower odds of RAR events in the pediatric inpatient setting, while high acuity locations may be at higher risk. Patient photographs may reduce WPOE without interruptions.

## Background

Computerized provider order entry (CPOE) systems are a ubiquitous component of the healthcare system that reduced some types of adverse medication events,^1^ but may paradoxically increase the risk of other types of errors^2–5^ such as wrong-patient order entry (WPOE), in which a provider intends to order a medication, lab, or procedure for one patient but unintentionally orders it for a different patient.^3^^,^^6^^,^^7^ The effects of WPOE range from harmless errors that never reach the patient to accidental death in the event of wrong-patient medication errors, though such dangerous complications are rare.^2^^,^^6^^,^^7^ Additionally, this important medical error is somewhat difficult to track given its rarity.^2^

The wrong patient retract-and-reorder (RAR) measure is a validated proxy measure with 76% positive predictive value (PPV) for detecting intercepted WPOE^2^ that is endorsed by the National Quality Forum. A measure of a near-miss event, RAR is an instance in which a provider retracts an order for a patient within 10 minutes of placing the order and then reorders the same order on a different patient within 10 minutes of the cancellation. Other studies have demonstrated that near-miss events have the same causal pathways as the adverse event,^8^^,^^9^ making it reasonable to assume that interventions lowering the RAR rate would also reduce WPOE. RAR events also occur often enough to capture and to track changes or improvements over time. While reported RAR rates vary drastically in the literature, the average rate is approximately 100 events per 100 000 orders.^1^^,^^3^^,^^10^^,^^11^ RAR events occur much more commonly in the inpatient setting than outpatient and are more common in acute care areas such as the ICU and emergency department.^3^^,^^4^ In prior studies, RAR events occurred at similar rates in the pediatric setting compared to adult settings, and pediatric emergency departments, ICUs, and NICUs are areas of particularly high risk.^10^^,^^12^

Strategies to reduce RAR events have focused on reducing multitasking or using interruptive approaches to assure patient identification. However, limiting the number of open charts did not reduce the RAR rate in a randomized controlled trial,^3^ and interruptive approaches that force providers to enter patient identifiers before placing orders or pop-ups to alert providers about similar names reduce RARs, but increase order-entry time and slow provider workflow.^7^^,^^13^ Additionally, multiple exposures to electronic alerts can decrease engagement with them, a phenomenon known as alert fatigue.^4^^,^^7^^,^^14^

One promising non-interruptive strategy is the display of patient photographs in the electronic health record (EHR). Patient photographs offer providers a visual reminder of the patient whose chart is open and have been associated with reduced WPOE in emergency department settings, without prolonging provider workflows. Additionally, patients understand the benefits of photo usage, and only very rarely refuse their usage due to concerns for their privacy.^15^^,^^16^ Patient photos in adult emergency departments have been associated with a lower risk of RARs,^7^ but this finding has not yet been applied in pediatric or inpatient settings, where RAR rates have historically been higher. In this retrospective cross-sectional study, we aimed to determine if the presence of a patient photo in the EHR was associated with a reduced risk of RAR orders in the pediatric inpatient setting.

## Methods

### Study design and setting

This study was performed from January 1, 2020 to July 1, 2022 in an urban, academic pediatric health system in the Southeastern United States with 3 freestanding children’s hospitals managing over 27 000 inpatient visits per year. The hospital system began a pilot program to attach patient photographs to the EHR on September 16, 2020 in 1 day surgery department and expanded on March 11, 2021 to registration staff across all 3 hospitals and main clinic locations, excluding emergency departments, urgent cares, and neighborhood clinics. Photographs immediately crossed to the EHR without being stored on the local computer or requiring manual file movements by the registration staff. Patients had the option to opt out of photos. This study was deemed non-human subjects research as part of a quality improvement initiative by the Children’s Healthcare of Atlanta IRB (STUDY00001628). Data are available upon request to the corresponding author.

### Outcome measure

The primary outcome for this study was RAR events as described above.^3^

### Statistical analysis

We used order sessions (instead of individual orders) as the unit of analysis for this study because (1) if one order of an order session is placed on the wrong patient, by definition all of the other orders will be as well, artificially increasing the RAR rate and the perceived benefit of patient photos and (2) the only randomized controlled trial focused on RAR event reduction used order sessions as the denominator.^3^ The primary outcome was a dichotomous variable of whether or not the order session represented an RAR event, reported as RAR events per 100 000 order sessions.

Our primary exposure was the presence of a patient photo in the EHR prior to the order session sign time. Additional covariates included characteristics of the patient (age, sex, race, ethnicity, insurance status, and the number of complex chronic conditions^17^), provider (Attending, PA/NP, Fellow, resident, or other), and ordering context (general care, cardiac, ICU, and other; day vs. night shift).

We calculated descriptive statistics and conducted 2-sided *t*-tests for continuous variables and Χ^2^ tests for categorical variables. To assess the effect of the photos on the overall RAR rate, we conducted 2-sided tests of equal proportions comparing the RAR rate during the pre-intervention period to the rate during the pilot period and during the full-go-live. To assess the relationship between patient photos and WPOE, we first conducted univariable logistic regression analyses with each of our covariates of interest as the independent variable and RAR session as the dependent variable. In the multivariable model, we included all covariates with *P *<.1 (all except complex chronic conditions). We also excluded values with small cell sizes (unknown sex [*n* = 51], and self-pay insurance [*n* = 5 among RAR events]).

As a sensitivity analysis, we performed a mixed effects multivariable logistic regression with random effects of the patient and provider ID to account for potential clustering. Finally, we determined factors associated with having a patient photo by creating a logistic regression model with the presence of a patient photo as the outcome and all other covariates from our primary analysis as predictors.

All analyses were conducted using R version 4.0.2 using the tidyverse and lme4 packages.^18–21^

## Results

**Figure 1.**
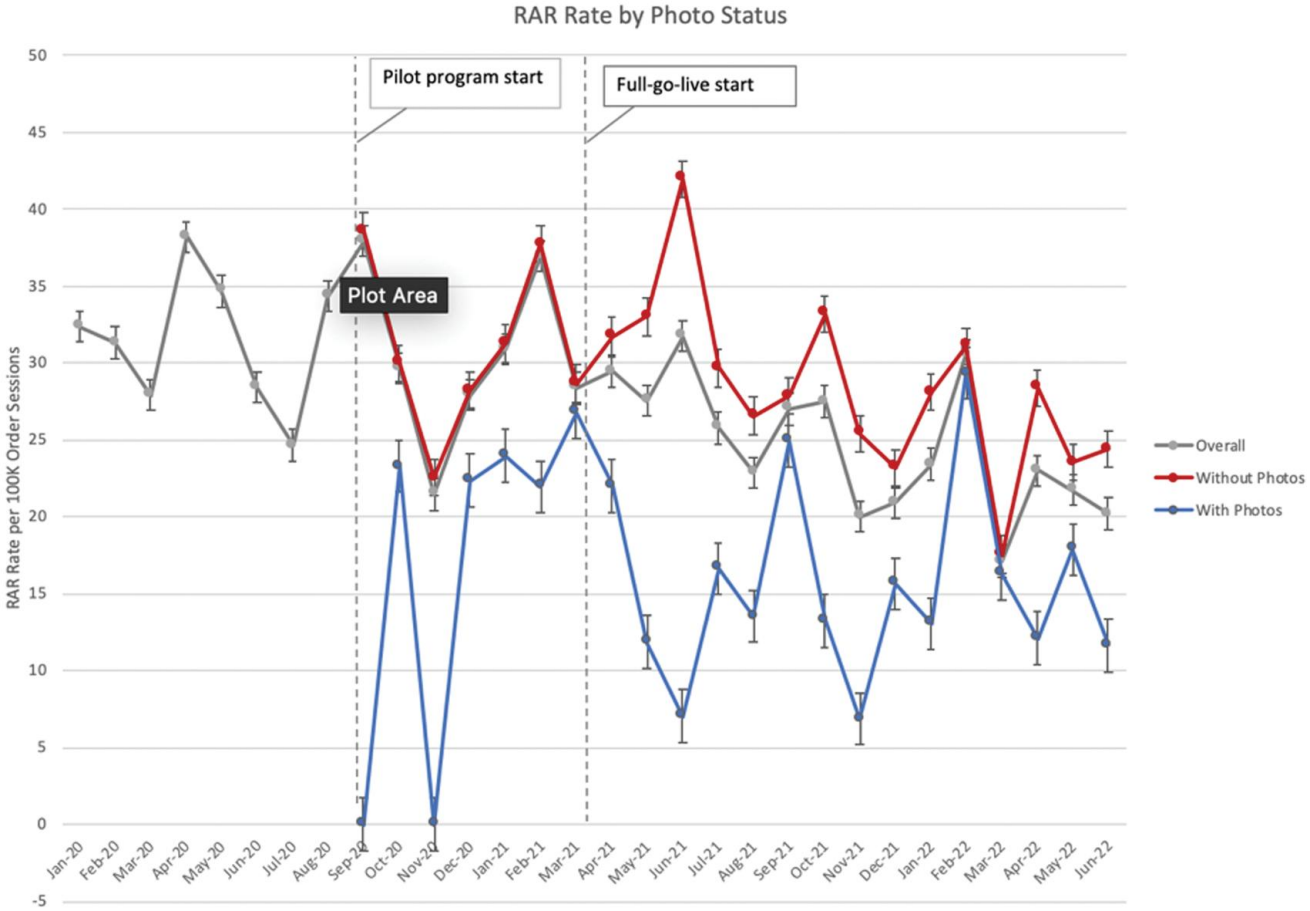
Retract and reorder (RAR) event rate over time including overall rate (gray) and stratified by patient photo status.

Our final sample included 85 767 unique patients for whom 3 414 770 order sessions were placed, 944 of which were RAR events in 886 unique patients (Table 1). The mean number of order sessions per patient was 40.2. At the time of each order session, 573 352 (16.7%) of patients had a photo in their chart. The overall RAR rate per 100 000 order sessions was 31.9 prior to patient photo implementation, 30.2 during the pilot phase, and 24.3 following the full-go-live, with the rate during the full-go-live representing a statistically significant decrease compared to the pre-intervention period (*P *= .56 [pre-intervention to pilot], *P* = .002 [pre-intervention to full-go-live]). The reduction in RAR rate was also temporally associated with the full-go-live (Figure 1).

**Table 1.** Order session characteristics by RAR session, 2020-2022.

	RAR session^a^		
Independent variables	No *N* = 3 413 826	Yes *N* = 944	*P* ^b^
Photo prior to order session			<.001
No	2 840 564 (78.6)	854 (90.5)	
Yes	573 262 (16.8)	90 (9.5)	
Age, years^c^ [mean (SD)]	7.19 (6.70)	5.75 (6.50)	<.001
Sex			.880
Female	1 619 408 (47.4)	445 (47.1)	
Male	1 794 367 (52.6)	499 (52.9)	
Unknown	51 (0.0)	0 (0.0)	
Race			
White	1 605 254 (47.0)	433 (45.9)	.498
Black	1 555 859 (45.6)	471 (49.9)	.009
Asian	129 776 (3.8)	33 (3.5)	.685
Other	21 552 (0.6)	8 (0.8)	.527
Ethnicity			.004
Hispanic	508 928 (14.9)	109 (11.5)	
Non-Hispanic	2 896 028 (84.8)	833 (88.2)	
Group			<.001
Cardiac	360 713 (10.6)	153 (16.2)	
General care	991 455 (29.0)	216 (22.9)	
ICU	941 701 (27.6)	379 (40.1)	
Other	1 119 957 (32.8)	196 (20.8)	
Shift			.064
Day	2 501 458 (73.3)	666 (70.6)	
Night	912 368 (26.7)	278 (29.4)	
Provider			.007
Attending	1 464 390 (42.9)	353 (37.4)	
Fellow	255 723 (7.5)	78 (8.3)	
Resident	646 791 (18.9)	210 (22.2)	
PA/NP	879 300 (25.8)	260 (27.5)	
Other	167 622 (4.9)	43 (4.6)	
Insurance			
Public	2 098 523 (61.5)	610 (64.6)	.051
Private	1 261 255 (36.9)	329 (34.9)	.194
Self pay	54 046 (1.6)	5 (0.5)	.014

a Count (column percentage); percentages may not add due 100 due to rounding.

b *P* values determined by Chi-square tests for categorical variables and *t*-tests for continuous variables.

c Age in years at time of order session.

In univariable analyses (Table 2), patient photos were associated with 48% lower odds of an RAR event (OR 0.52, CI 0.42-0.64). Age and Hispanic ethnicity were also associated with lower odds of an RAR event (OR 0.96 per year, CI 0.95-0.97; OR 0.74, CI 0.61-0.90, respectively). Service groups (general care, cardiac, and ICU, compared to other groups), public insurance (compared to private), and provider type (resident and PA/NP, compared to attending) were all associated with higher odds of an RAR session, with service groups having the largest effect sizes.

**Table 2.** Association of patient photos and other control variables with odds of RAR events in univariable and multivariable analyses.

Independent variables	OR	95% CI	aOR	95% CI
Photo prior to order session				
No	Ref			
Yes	**0.522**	**0.420-0.648**	**0.604**	**0.484-0.754**
Age, years	**0.966**	**0.956-0.976**	**0.985**	**0.974-0.995**
Sex				
Female	Ref			
Male	1.01	0.89-1.15	0.995	0.875-1.13
Race				
White	0.954	0.839-1.08	**1.30**	**1.00-1.70**
Black	**1.18**	**1.04-1.35**	**1.32**	**1.00-1.74**
Asian	0.916	0.647-1.29	1.16	0.769-1.77
Other	1.34	0.670-2.69	1.55	0.768-3.16
Ethnicity				
Non-Hispanic	Ref			
Hispanic	**0.745**	**0.610-0.909**	**0.802**	**0.642-1.00**
Group				
Other	Ref			
Cardiac	**2.42**	**1.96-2.99**	**2.12**	**1.69-2.67**
ICU	**1.24**	**1.02-1.51**	**2.05**	**1.71-2.45**
General care	2.29	1.93-2.73	1.13	0.934-1.39
Shift				
Night				
Day	0.873	0.759-1.00	0.881	0.764-1.01
Provider				
Attending	Ref			
Fellow	1.26	0.990-1.61	1.06	0.825-1.51
Resident	**1.34**	**1.13-1.59**	**1.37**	**1.15-1.63**
PA/NP	**1.22**	**1.04-1.43**	1.00	0.852-1.19
Other	1.06	0.775-1.46	1.10	0.804-1.51
Insurance				
Private	Ref			
Public	1.14	0.974-1.27	1.09	0.949-1.26

Bold values indicate statistical significance (*P* < .05).

In multivariable analysis (Table 2), the association between patient photos and lower odds of an RAR event persisted (aOR 0.60, CI 0.48-0.75). Each 1-year increase in age was associated with lower odds of an RAR event (aOR 0.98, CI 0.97-0.99). Cardiac (aOR 2.12, CI 1.69-2.67) and ICU (aOR 2.05, CI 1.71-2.45) service groups were associated with higher odds of RAR sessions compared to other service groups. Finally, resident provider was associated with higher odds of RAR session (aOR 1.37, CI 1.15-1.63) compared to attending providers.

In multivariable analysis examining factors associated with having a patient photo in the chart prior to hospital discharge (Table S1), Black patients had lower odds of having a patient photo compared to White patients (aOR 0.89, CI 0.87-0.93). Additionally, Hispanic patients had lower odds than non-Hispanic patients of having a patient photo (aOR 0.77, CI 0.73-0.81). Patients admitted to the cardiac, ICU, or general care setting also had lower odds of having a patient photo (aOR 0.59, CI 0.55-0.64; aOR 0.45, CI 0.43-0.47; aOR 0.44, CI 0.42-0.45, respectively). Finally, patients admitted during the day shift (compared to night) had higher odds of having a patient photo (aOR 1.35 CI 1.31-1.40). Each 1-year increase in age was associated with higher odds of having a patient photo (aOR 1.05 CI 1.04-1.06).

Sensitivity analyses utilizing patient or provider ID as mixed effects to account for potential clustering produced very similar results to the multivariable logistic regression reported above (Table S2).

## Discussion

We found that having a patient photograph in the chart was associated with a 40% reduction in the adjusted odds of an RAR event. Additionally, we found lower RAR rates after system-wide implementation of an effort to add patient photos to the chart. Our results support the findings of other studies conducted in the adult population and suggest that patient photographs are an effective tool to reduce WPOE in the pediatric population, including in the general inpatient setting.

Higher acuity care settings (cardiology, ICU) had an outsized association with RAR events in our study, perhaps due to more frequent order sessions, task switching, and interruptions in these environments. In Adelman et al, the RAR rate was similarly much higher in the critical care setting with >250 RAR events per 100 000 order sessions when averaged across both trial groups.^3^^,^^22^^,^^23^ Additionally, we observed a small but statistically significant association between increased age and decreased RAR events, which may be related to the development of more distinct distinguishing features as children age. For example, RAR rates are higher in neonatal ICUs compared to other pediatric settings.^10^^,^^12^ Finally, that patients of Black race, Hispanic ethnicity, and those cared for in the cardiac, ICU, or general care settings had lower odds of having a patient photo warrants attention. The reason for these associations is unclear, but one possibility is that patients who are more likely to be seen in ambulatory or non-acute care settings may be more likely to have patient photos taken, as capturing photos fits more easily into the workflow in these outpatient settings. While it is beyond the scope of this study to determine the mechanism, these findings provide evidence that the process for obtaining patient photos may lead to structural inequities.

While RAR rates in pediatric settings have been lower than adult settings in prior studies,^4^ the overall RAR rate even prior to implementation of patient photos at our institution (31.9 per 100 000 order sessions) was substantially lower than the RAR rate reported in other studies for an unknown reason.^3^^,^^6^^,^^7^ The code used to extract RAR events was previously validated and publicly available here for our EHR vendor (Epic Systems^©^),^24^ so it is less likely that the observed RAR rate is due to a data extraction error. Nonetheless, the lower RAR rate at our institution would have biased results toward the null hypothesis of no effect of patient photos; observing an effect size commensurate with other studies provides support for the effectiveness of patient photos to reduce WPOE, even in settings with low baseline RAR rates.

Our study has several limitations. It was conducted at a single urban, pediatric academic health system in the inpatient setting and findings may not be generalizable to other settings. Second, the cross-sectional nature of the study limits the ability to draw conclusions regarding causation, but the ability to view trends in the overall RAR rate over the course of the implementation of patient photos provides some evidence about the effectiveness of patient photos. We are also unable to verify if or how providers may have consciously or unconsciously looked at patient photos to verify identity before signing orders. However, prior studies have used eye-tracking capabilities to determine that providers primarily use the banner to verify patient identity in the EHR, which increases the plausibility of the mechanism of patient photos to reduce RAR events. Third, since photos were not collected for patients <6 months of age, it is unclear how effective patient photos would be to prevent RAR events in a setting where patients have similar demographic characteristics and fewer distinguishing features. Finally, it is possible that the observed effect of patient photos is due to unmeasured factors, such as provider interruptions, multitasking, or the nature or purpose of particular orders or service groups or to measured factors such as the lower rate of patient photos in certain demographic groups. However, given the persistent, robust relationship between patient photos and RAR events in our multivariable model even after accounting for race and ethnicity, we believe it unlikely that the association between photos and RARs can be fully accounted for by confounding from any measured factors including demographics.

We found that patient photos in the EHR were associated with 40% lower odds of RAR events in a pediatric inpatient setting. This effect size is consistent with prior studies and suggests that efforts to increase patient photo adoption may reduce WPOE and associated safety events. Additional research is needed to elucidate the effect of patient photos on WPOE and to define strategies that improve patient photo uptake in the EHR.

## Supplementary Material

ooae042_Supplementary_Data

## Data Availability

The data underlying this article will be shared on reasonable request to the corresponding author.
