# Supplementary material for: Association of patient photographs and reduced retract-and-reorder events
Source: JAMIA Open. 2024 Jul 2;7(3):ooae042. doi: 10.1093/jamiaopen/ooae042 (PMC11218880; doi:10.1093/jamiaopen/ooae042)
Supplement: ooae042_Supplementary_Data [file ooae042_supplementary_data.pdf]

Supplemental Table I: Association of patient photos and other control variables with odds of RAR events in mixed-effects models with provider and patient entered as random effects

	Patient		Provider	
Independent Variables	aOR	95% CI	aOR	95% CI
Photo prior to order session				
No	Ref			
Yes	0.604	0.484-0.755	0.605	0.484-0.757
Age, years	0.985	0.975-0.995	0.984	0.973-0.995
Sex				
Female	Ref			
Male	0.995	0.874-1.13	0.995	0.875-1.13
Race				
White	1.30	1.00-1.70	1.30	1.00-1.70
Black	1.32	1.00-1.74	1.32	1.00-1.74
Asian	1.16	0.769-1.77	1.17	0.773-1.78
Other	1.54	0.757-3.16	1.55	0.768-3.16
Ethnicity				
Non-Hispanic	Ref			
Hispanic	0.803	0.641-0.1.00	0.805	0.644-1.00
Group				
Other	Ref			
Cardiac	2.12	1.68-2.67	2.08	1.62-2.67
ICU	2.05	1.71-2.45	2.04	1.68-2.45
General Care	1.14	0.935-1.39	1.13	0.919-1.39
Shift				
Night				
Day	0.881	0.765-1.01	0.918	0.793-1.06
Provider				
Attending	Ref			
Fellow	1.06	0.823-1.36	1.02	0.767-1.37
Resident	1.37	1.15-1.63	1.38	1.13-1.68
PA/NP	1.00	0.852-1.19	0.981	0.807-1.19
Other	1.10	0.804-1.51	1.12	0.797-1.59
Insurance				
Private	Ref			
Public	1.09	0.949-1.26	1.08	0.944-1.25

Supplemental Table 2: Predictors of the presence of patient photos

Independent Variables	AOR	95% CI
Age, years	1.06	1.06-1.06
Sex		
Female		
Male	1.01	1.01-1.02
Race		
White	1.01	1.00-1.02
Black	0.87	0.86-0.88
Asian	0.99	0.97-1.02
Other	0.62	0.59-0.65
Ethnicity		
Non-Hispanic		
Hispanic	1.01	1.00-1.02
Group		
Other		
Cardiac	0.48	0.47-0.49
ICU	0.48	0.48-0.48
General Care	0.54	0.53-0.54
Shift		
Night		
Day	1.29	1.28-1.30
Provider		
Attending		
Fellow	1.61	1.59-1.63
Resident	1.72	1.70-1.73
PA/NP	1.60	1.59-1.61
Other	0.99	0.99-1.02
Insurance		
Private		
Public	1.05	1.04-1.06
